# Help us make a better flow in everyday life - family needs in home-based pediatric palliative care: a qualitative study

**DOI:** 10.1186/s12904-025-01881-5

**Published:** 2025-09-26

**Authors:** Linda J. Martinsen, Simen A. Steindal, Heidi Holmen, Anette Winger

**Affiliations:** 1 Faculty of Health Sciences, Department of Nursing and Health Promotion, Oslo Metropolitan University, Postbox 4, St. Olavs Place, Oslo, NO-0130 Norway; 2 VID Specialized University, Oslo, Norway; 3 Lovisenberg Diaconal University College, Oslo, Norway; 4 Oslo University Hospital, Oslo, Norway

**Keywords:** Pediatric palliative care, Home-based care, Family needs, Communication, Coordination, Qualitative study

## Abstract

**Background:**

Pediatric palliative care focuses on optimizing the quality of life for children with life-limiting or life-threatening conditions and their families. Home-based pediatric palliative care is often recommended as the preferred model of care. To ensure effective home-based care, it is essential to deepen our understanding of the specific needs of families in this context. This study aimed to explore family care and communication needs in home-based pediatric palliative care from the perspective of the parents.

**Methods:**

This qualitative exploratory study was conducted with five focus groups with a total of 18 parents of children with life-threatening or life-limiting conditions. Data were analyzed using narrative thematic analysis.

**Results:**

Four main themes were developed: (1) The healthcare system lacks a comprehensive understanding of family needs; (2) A dedicated coordinator can alleviate the caregiving burden on parents; (3) A hotline to healthcare professionals familiar with the child is essential; and (4) A shared health record system can enhance information sharing and provide parents with a sense of control and oversight.

**Conclusion:**

Parents of children with life-limiting or life-threatening conditions face complex caregiving responsibilities while simultaneously navigating the healthcare system, seeking information, and coordinating care. These findings highlight the importance of a dedicated care coordinator in communicating with the various healthcare services involved. Also, parents emphasized the need for streamlined information sharing and easier access to critical health information regarding their child’s condition.

**Supplementary Information:**

The online version contains supplementary material available at 10.1186/s12904-025-01881-5.

## Background

Globally, approximately 21 million children are estimated to be eligible for pediatric palliative care (PPC) [1]. Based on international estimates [1, 2], approximately 8000 children in Norway live with life-limiting and/or life-threatening (LL/LT) conditions and are eligible for palliative care [2, 3]. PPC aims to ensure the best possible quality of life (QoL) for both the child and the child’s family by providing comprehensive support that addresses physical, psychological, social, spiritual, and existential needs [4]. PPC is recommended for children aged 0–18 with LL/LT conditions, a heterogeneous group that includes both cancer and non-cancer conditions [1]. PPC should be initiated at the time of diagnosis and continue throughout the illness trajectory [1, 2].

Advancements in medicine and healthcare have extended the lives of many children with LL/LT conditions, thereby increasing the need for long-term and comprehensive care [1, 2]. These children often experience changing and complex needs throughout their illness, and their needs encompass medical, personal, and social dimensions [5–7]. For some children, it is particularly important to encounter healthcare professionals (HCPs) who take the time to get to know them, provide clear and sufficient information, and involve them meaningfully in decision-making related to their condition and care [7].

Parents value having a clear understanding of their real options of care, effective communication and support from HCPs in home-based settings, while also managing the needs of siblings and maintaining overall family life [7, 8]. Collaboration between primary and specialist healthcare services involved in PPC is key to ensuring high-quality care, effective communication, and coordination [9, 10]. Despite notable variation in how PPC is delivered, both across countries and within comparable regions such as the Nordic countries [3, 6], home-based PPC is often recommended as the preferred model of care [1]. However, access to and implementation of home-based PPC varies significantly depending on healthcare system structures, available resources, and individual needs. Home-based PPC can take various forms, and in the Norwegian context it typically involves support from the healthcare services the child already receives, including both primary and specialist care [3], well aligned with the international consensus [1]. In some cases, this is supplemented by services from advanced home hospitals for children and/or hospital-based PPC teams [3]. Some families maintain regular contact with home-based teams, some of them providing 24/7 support, while other families receive support from both these teams primarily during periods of complex treatment and/or in the terminal phase of the child’s illness, with individual adjustments regarding the team’s availability [3]. The decision to remain at home is not universally preferred or feasible; children and families may require specialist care in hospital settings depending on the phase of illness and the complexity of needs [7, 11]. This follow-up enables children to remain at home for as long as possible, thereby reducing the burden of frequent hospital visits while ensuring access to necessary medical treatment and care [9]. Although home-based care can be challenging and may provoke uncertainty and fear, many parents prefer to remain at home with their child for as long as possible [6, 7, 12]. Families value the normalcy and familiarity of the home environment, which provides a sense of security, strengthens family bonds, and supports the continuity of everyday life [13–15]. Nevertheless, while home-based PPC requires the involvement of a skilled and coordinated care team [7, 16], studies consistently highlight parents’ experiences of poorly coordinated services and communication gaps with HCPs [10, 15].

To facilitate successful home-based PPC, it is essential to understand the needs of families and the conditions under which care is delivered [9, 17, 18]. However, there remains a significant knowledge gap regarding the specific needs of parents providing home-based care to children with a wide range of LL/LT conditions across different phases of the PPC trajectory, not solely during end-of-life care, which has been the primary focus of much existing research [7, 12, 17]. Furthermore, limited research has examined the perspectives of diverse families and explored the care and communication needs of not only ill children but also their parents and siblings. Developing such knowledge is crucial for informing clinical practice and delivering tailored care that supports both the child and their family, while also acknowledging and strengthening their role in the caregiving process [19, 20]. In response to these gaps, this study aimed to explore the families’ care and communication needs in home-based PPC from the perspective of parents. Specifically, it addressed the research question: *How do parents of children with LL/LT conditions perceive the care and communication needs for home-based PPC?*

## Methods

### Design

This study employed a qualitative exploratory design, using focus groups to explore parents’ care and communication needs in home-based PPC. Focus groups were chosen to leverage the value of participant interaction and collective discussion, which can elicit insights that differ from those obtained through individual interviews. While individual interviews often yield in-depth personal narratives, focus groups allow for the exploration of shared experiences, attitudes, and perspectives, particularly relevant for topics that affect participants in similar ways [21]. This method was considered appropriate given our aim to understand commonalities in how parents experience and perceive care and communication needs [21]. This study was guided by a family-centered theoretical framework, which emphasizes collaboration with families throughout the care process, acknowledges their expertise, and promotes individualized support that addresses the holistic needs of both the child and the family [1, 19].

This study explores a research question within the research project *Health Technology in Home-based Pediatric Palliative Care (CHIP homeTec)*, which explores key aspects relevant to the design of digital solutions aimed at supporting communication among children, families, and healthcare personnel involved in home-based PPC. The present study focuses specifically on the needs of families, as identified through a series of focus group discussions. This article has been reported in accordance with the Consolidated Criteria for Reporting Qualitative Research (COREQ) guidelines [22, 23].

### Participants

Parents of children living with LL/LT conditions were recruited through an open online survey conducted as part of the CHIP homeTec project, in which participants could indicate their willingness to be contacted for focus group participation. In addition, eligible participants were recruited for a pilot focus group at a specialized habilitation outpatient care services for children with LL/LT conditions. Purposeful sampling was employed based on the following inclusion criteria: parents of children aged 0–18 years with an LL/LT condition, residing in Norway, and able to participate in a Norwegian language focus group. We did not develop specific exclusion criteria as the inclusion criteria were mutually exclusive, that is, if they did not have a child eligible for PPC, they were not eligible for participation. Parents who expressed interest in receiving more information were contacted by LJM via email or phone and provided with both written and oral information about the study. A total of 25 parents consented to participate. However, six were unable to participate due to acute illness (either their own or within the family) or other unforeseen personal circumstances. One parent withdrew without providing a reason. The final sample consisted of 18 parents of children aged 1.5 to 18 years, representing all four Norwegian health regions. The participating parents had children with various medical conditions, all of which met international criteria for eligibility for PPC [1], and represented three of the five categories of LL/LT conditions (see Table 1). The children and their families represented in this study received various types of healthcare services to meet their PPC needs. Some were in contact with palliative care teams within primary and/or specialist healthcare services, while others already had established healthcare services tailored to the child and family’s existing needs.

**Table 1:** Demographic characteristics of parents (*N* = 18) and their ill children (*N* = 14)

Characteristics of the parents and children	
Parent characteristics	**N = 18​**
Age parent, median ​(min–max)​	​40.0 ​ (29–49)​
Gender parents, female, n (%)​	​15 (83)​
Child characteristics	**N = 14**
Age child, median ​(min–max)​	​5.8 ​ (1.5–18.0)​
Gender child, female, n (%) ​	​8 (57)
Children with siblings, n (%)	11 (79)
Health conditions of children ^a^	
Life-threatening conditions	3 (21)
Conditions where premature death is inevitable	-
Progressive conditions without curative treatment options	4​ (29)
Irreversible but nonprogressive conditions	5 (36)
Unborn children with major health problems	-
Others ​(combined conditions compatible with PPC)	2 (14)
Norway Regional Health Authority (RHA) regions	
Northern Norway RHA​	4​ (29)
Central Norway RHA​	1​ (7)
Western Norway RHA	7​ (50)
South-Eastern Norway RHA	2 (14)

^a^. Categories of LL/LT conditions: (1) Life−threatening conditions for which curative treatment may be feasible but can fail; (2) Conditions in which premature death is inevitable; (3) Progressive conditions without curative treatment options; (4) Irreversible but nonprogressive conditions causing severe disability leading to susceptibility to health complications and the likelihood of premature death; and (5) Unborn children who may not live through birth, infants who may survive for a few hours/days, infants with birth anomalies or for whom intensive care has been appropriately applied but developed an incurable disease (1)

### Data collection

An interview guide with open-ended and probing questions was developed to facilitate reflection and discussion within the focus groups. A reference group reviewed the guide to ensure that the questions were clearly formulated and relevant. The reference group included user representatives such as parents of children with LL/LT conditions and representatives from various user organizations, and HCPs and researchers with experience from the PPC field. The interview guide was pilot-tested and subsequently revised with minor adjustments to wording and question order. The final version included questions about parents’ views and experiences with healthcare services, home-based care, and health technology (Supplementary File 1). The guide also addressed the everyday needs of the parents, the ill child, and the siblings, as well as how those needs could be met, aligning with the family centered theory [19] at the core of the current project.

A total of five focus groups, including the pilot interview, were conducted between December 2023 and March 2024. Each focus group consisted of 3–5 parents. In some groups, both parents of a child participated, while in others, either the mother or the father attended alone. Each interview lasted from about 50 to 85 min (median: 65 min), with no breaks or interruptions. To ensure that the participants felt comfortable and to avoid triggering memories or associations with places such as hospitals, the focus groups were held in neutral locations, such as meeting rooms in hotels. Three focus groups were conducted in person, while two were held online via Microsoft Teams, using both audio and video features. However, only the audio portion of the interviews was recorded. The first author acted as the moderator (LJM) in all groups, while one of the coauthors acted as the assistant moderator (AW or SAS). The moderators presented themselves and their professional backgrounds and experiences. Each focus group began with an introduction to the project, followed by an explanation of the focus group’s aim, which was to explore family care and communication needs. All focus groups were audio recorded, and the encrypted recorded material was digitally transferred to the Service for Sensitive Data for secure storage, transcription, and analysis, only available to the four authors of this article [24].

### Analysis

The first author transcribed the recorded material from the focus groups. NVivo software (QSR International Pty Ltd., Victoria, Australia) was used to organize and manage the data. The data material was analyzed using narrative thematic analysis, which combines elements from both narrative analysis and thematic analysis to examine stories and identify underlying themes [25]. As described by Riessman [25], we focused on the content of the text—that is, “what” was being said, rather than “how” it was said. We emphasized the underlying themes in the narratives and how these themes gave meaning to the experiences being told. Stories (or narratives) are defined as structured accounts that people create to make sense of their experiences. These narratives are shaped by the context in which they are told and by the intended audience [25].

The interviews were read repeatedly to become thoroughly familiar with the data, and notes on emerging ideas or reflections were written directly on printed copies of the transcripts. A systematic inductive coding process was then initiated to identify patterns in the participants’ stories relevant to the research question. These patterns were drawn from sentences, paragraphs, and complete stories. Initial themes were developed by manually grouping codes using NVivo based on identified patterns across the dataset. The themes underwent several review rounds, during which the codes were reorganized, expanded, or narrowed down. Preliminary themes were created, and the authors collaborated to enhance, clarify, and label the final themes. At the end of the analysis process, all data were reviewed to identify stories supported by parents’ quotes that illustrated the final themes, and the results were subsequently documented. The analysis was conducted by LJM in collaboration with the three co-authors (AW, HH, and SAS). The authors met several times to reflect on and discuss all phases of the analysis process, gaining new insights and providing alternative interpretations, which is the essence of reflexivity in the analysis process [26].

### Preunderstanding

All authors have professional backgrounds as HCPs. LJM is a Ph.D. candidate and pediatric occupational therapist with 15 years of experience in specialist habilitation care for children with and without a need for PPC. This includes experience working with children with LL/LT conditions, which has given insight into the emotional and practical challenges families face. This experience has shaped a deep understanding of the importance of family involvement in caregiving. HH, SAS, and AW, are all registered nurses and have clinical and/or considerable experience in qualitative research in pediatrics, palliative care, critically ill patients, and health technology. All authors are members of the national research network “Children in Palliative Care” (CHIP) and are engaged in the CHIP homeTec research project. To address potential biases, we have actively sought to include diverse perspectives from each other and have been mindful of reflecting on how our preunderstanding affects the research process.

### Ethical considerations

The study obtained ethical approval from the Regional Committees for Medical and Health Research Ethics in Norway (reference number 251065) and the Sikt - Norwegian Agency for Shared Services in Education and Research (reference number 657413). All participants received oral and written information about the aim of the study and the possible benefits and consequences expected from participating. They were informed that participation was voluntary and that they could withdraw at any time. They were informed about the confidential handling of all personal data. Before participating in the focus groups, digital informed consent was obtained from all participants. According to the ethical approval, we were not allowed to collect data regarding the duration of the children’s condition, receipt of palliative care or similar aspects.

## Results

Following the analysis, four themes were developed illustrating the care and communication needs described by the parents (see Table 2): (1) The healthcare system lacks a comprehensive understanding of family needs; (2) A dedicated coordinator can alleviate the caregiving burden on parents; (3) A hotline to healthcare professionals familiar with the child is essential; and (4) A shared health record system can enhance information sharing and provide parents with a sense of control and oversight.

**Table 2:** Examples of the analysis process and theme development

Themes	Codes	Excerpts from related stories/quotes
The healthcare system lacks a comprehensive understanding of family needs	• Their own needs to be taken care of Fight for the rights of their child and the family • Take care of complex nursing and care tasks • Lack of understanding of the family’s overall needs • Siblings need follow-up	… We actually reached out to Child Protective Services once, to say that we needed help. They gave us someone to talk to, and when we finally got started and began to benefit from it, it was taken away. Because then we got Personal Assistance, and everything was supposed to be fixed. So we kind of lost that person, and then we’re left standing a bit on our own … The focus is on what’s best for the child, but what’s best for the parents is what’s best for the child … (Participant 2, FG 2)
A dedicated coordinator can alleviate the caregiving burden on parents	• The coordinator does not give parents real relief from their duties • Lack of collaboration creates additional work for the parents • Coordinators who function their duties are an invaluable help • Wish for a coordinator in the specialist health service More coordinated planning of the child’s follow-up	… The first thing I thought of when they said coordinated services, which would make our daily lives a hundred times easier, was if the different departments and doctors could talk to each other and coordinate things when we go to the hospital… But then they only set up one appointment, if they were a bit proactive, they could get several things done at the same time by coordinating it. That would save us an incredible amount of travel days. It’s one thing for us parents to travel, but for the kids in all of this who are sick and have their own problems, it’s quite a big strain for them with travel, hospitals, and people in white coats and blue gloves. So that’s one of the biggest wishes we have for the hospitals, this is priority number one for us … (Participant 2, FG 4)
A hotline to healthcare professionals familiar with the child is essential	• Need to confer with HCPs in different situations • Values the follow-up at the hospitals • Good contact persons are valued • Lack of competence about the child in the primary healthcare services • Variety in availability and follow-up from HCPs • Values contact with HCPs who have expertise in the child’s condition • Easy and direct contact with HCPs	… For the diabetes, it’s been absolutely great, both the system and having an open line to the Children’s Ward at the hospital. So if there’s ever something urgent, we can call there and get good answers right away. So it’s just been, that follow-up has been incredibly … (Participant 2, FG 2)
A shared health record system can enhance information sharing and provide parents with a sense of control and oversight	• Try to keep track of information • Lack of available information and overview • Simplify solutions for information sharing • Wish to gather all information about the child • Wish for a shared electronic health record around the child	… We’ve mostly acted as the middlemen, passing along what the hospital wants us to communicate to the home care service and the doctor’s office. So we’ve kind of taken responsibility to make sure they get the information, but I wish there was a shared platform where, for example, the health nurse could communicate with the specialist health services about things like which vaccines he should get. Or what kind of follow-up we should provide him moving forward … That you could find the information you need in the same place… (Participant 2, FG 3)

*FG *focus group, *HCP* healthcare personnel

### The healthcare system lacks a comprehensive understanding of family needs

Parents described how living with a child with an LL/LT condition had a great impact on everyday life for the entire family. The parents experienced a lack of understanding about the needs of the family, and that the holistic approach to care was missing from the healthcare services they met. Parents described an almost constant struggle, with a lack of acknowledgment and understanding from both HCPs and healthcare systems. This resulted in frustration and, ultimately, a lack of or delayed access to relevant care services. Parents’ needs extended beyond addressing the medical care of their ill child and advocated for a more holistic approach. A holistic approach included elements such as respite care that allowed them moments of relief at home, the provision of essential technical equipment to support their child’s well-being, and access to financial assistance or care allowances that allowed the parents the possibility to care for their child at home. When one of their needs was met, the parents experienced that another service could be removed or changed or that the requested service was misinterpreted and backfired on them. One participant elaborated on how they lost access to support from child protective services when they were granted access to a personal assistant, even though their mandates differed.

Parents often found themselves in a constant balancing act between multiple roles, transitioning from being caregivers to advocates for their children. They frequently faced ongoing challenges when dealing with various healthcare services, striving to convey their family’s need for essential care. These interactions often brought about frustrating feelings, as they navigated a system they felt lacked an understanding of the complex circumstances of the family, and some parents described the system as not prepared for the complex care needs of their child.

*… When our son was diagnosed with this condition*,* a doctor told us that now we had to be his advocate*,* roll up our sleeves*,* and fight for him*,* because nothing comes on its own. And she was right. You feel like you have to push and push everywhere* … (Participant 1, FG 4).

Many parents longed for a parental role where they were allowed to be just parents. Instead, they felt more like full-time employees in their child’s care, rather than simply being mother and father. All the tasks and roles the parents were obliged to fulfill negatively affected their ability to take care of their well-being, and their relationships as husband and wife, and many could not obtain their professional work duties. One of the most negative consequences highlighted by parents in all interviews was the despair of not being sufficiently able to care for siblings. Parents often felt torn between the ill child and their other children, frequently resulting in siblings being sidelined. Parents lacked sufficient support to care for siblings, and they sought help and emotional support to care for the siblings’ needs. Like the fight described to receive the warranted care for the ill child, the parents described how they strived for support for the siblings as well. Parents having experiences with support services for siblings described them as highly valuable.

Descriptions of certain activities, including support conversations, meetings with others who share similar experiences, and sibling groups, were particularly valued in helping parents navigate their challenges managing their heavy everyday lives. Parents emphasized that these services were often something they had to request themselves.

### A dedicated coordinator can alleviate the caregiving burden on parents

The need to coordinate the child’s services was, at times, a consuming task for the parents, described as a burden that took their time away from being just a parent. They expressed frustration concerning the lack of coordinated consultations in hospitals, which ultimately placed a burden on both the child and the family. Coordinating various hospital appointments for their children became a time-consuming task, leading to significant distress and frustration. The parents described considerable shortcomings regarding interaction and coordination within and between specialist healthcare services. This included a lack of interaction across health regions but also within each local hospital. The parents’ stories exemplified this by illustrating how the hospitals scheduled several appointments in different departments at the same hospital over the course of a week, rather than clustering them all in one day. Often, appointments at the same hospital or consultations across different hospitals overlapped, forcing parents with the burden to spend time prioritizing and rescheduling consultations. When the parents coordinated appointments themselves, this reduced the parents’ absence from work and minimized the child’s absence from school or kindergarten, thereby causing less disruption to the child’s everyday life and routines, making the time-consuming organization worth the effort.

Most families had assigned coordinators or child coordinators in the municipal services. Still, the parents described how the coordinators lacked the mandate to perform the coordinating tasks that the parents needed them to do. According to the parents, the coordinators had too little time and scant resources. The parents felt that municipal coordinators could not help them nor provide sufficient support, as they could not manage coordination with or between different levels of specialist healthcare services.

*… We want them to coordinate and keep track at the hospital*,* but they’re not allowed to*,* and it just stops. And the people at the hospital don’t know what the child coordinator is supposed to do; it’s just a mess. And that’s why we parents end up being the coordinators and doing these things ourselves …* (Participant 1, FG 5).

The parents desired to limit the number of consultations and hospital visits for their children. This was important, as the parents emphasized maintaining relationships with friends and family to avoid disruption in routines and everyday life, as well as absence from school or kindergarten. Consequently, the parents emphasized the need for a more holistic approach to coordinating healthcare services for children with LL/LT conditions.

Parents suggested establishing a system where coordinators have a clear mandate and sufficient resources to manage all aspects of the child’s healthcare services. This included ensuring that all appointments and follow-ups were well-coordinated between various levels of the healthcare systems, allowing parents to focus more on caregiving and less on time-consuming coordination tasks.

### A hotline to healthcare professionals familiar with the child is essential

The parents expressed a strong need for accessible and available HCPs with whom they could easily and directly communicate, especially in critical situations. Parents spent a great deal of time trying to reach HCPs and hospital departments to get assistance or to voice medical concerns about their children. They experienced how HCPs in primary healthcare services or small local hospitals often fell short in several areas, such as issuing prescriptions, adjusting medications, discussing the adverse effects of medications, observing the child, and making necessary adjustments to the child’s care, which were mentioned as pressing issues and not easily resolved because the children had unique or rare care needs.

*… Availability from healthcare personnel. As I mentioned*,* they are available locally. We have open returns*,* it’s safe … But then*,* since the local hospital often falls short when it comes to very complex epilepsy*,* for example. And there I feel like there are gaps that are hard to fill*,* that you get enough help at the moment you need it … You don’t get the same answers locally*,* or they no longer dare or have to consult. So for us*,* it’s a wish for more availability from the specialist hospital. We know he has his doctor there … But at the same time*,* you often have to call the switchboard and you don’t necessarily get through quickly …* (Participant 1, FG 3).

From the parents’ stories, a close relationship with specialist healthcare services was frequently mentioned.

Parents valued meeting HCPs who possessed expertise about the child’s condition and diagnoses. Parents often received better support and had more opportunities to discuss their child’s condition with trusted HCPs at regional or larger hospitals. In contrast, parents expressed that primary healthcare services typically lacked the experience and expertise to address the concerns of parents effectively. Furthermore, the children had, and required, treatment in many departments within each hospital and at various levels of the health services, and the parents missed having dedicated HCPs who followed up and maintained an overall medical and treatment overview. While some children had designated pediatricians at the hospitals, parents expressed that the effectiveness of contacting them and the feasibility of maintaining an overview varied in practice. Parents who had a dedicated pediatrician or another dedicated HCP described this support as of great importance and enhanced the parents’ sense of security and support.

### A shared health record system can enhance information sharing and provide parents with a sense of control and oversight

The parents highlighted a need for an overview of their child’s follow-up of the care, medical history, and current medical status in everyday and critical situations. Their daily tasks included, for instance, obtaining documentation and sharing medication adjustment plans or wound care procedures with HCPs. Conveying necessary and critical medical information to ambulance personnel during a respiratory arrest or a severe epileptic seizure was another example of the importance of the need for easy, available information, as highlighted by the parents. The parents shared stories from situations in which they acted as a coordinating link between two hospitals during emergencies. This has made it crucial for the parents to keep track of the medications that have been administered and to remember a significant amount of medical history. The parents highlighted that this control and overview was extremely important, as no one can expect a doctor to review all the medical summaries for many years. Therefore, parents emphasized the prerequisite of having a medical history readily available.

The electronic health record (EHR) systems used in the children’s health services varied somewhat within the specialist healthcare service, while there was significant variation in EHRs used in primary healthcare systems. Parents reported a lack of communication between these EHRs, especially across service levels.

*… I think we have a few too many platforms that don’t work very well … If everything was gathered in one place*,* instead of documenting in so many places*,* it might work better … I think that’s a bit hard to achieve in a country as long as ours. But of course*,* if everyone used the same system across Norway and things were the same everywhere*,* that would be nice. That you could go to a national service and they find what they need*,* or if you move*,* you don’t have to start over …* (Participant 3, FG 4).

The parents emphasized the need for a shared EHR, which was anticipated to simplify the time-consuming work parents did to gather and share necessary information with HCPs and healthcare services involved with their child. This included sharing valuable information about everything from day-to-day care to more specific medical procedures and interventions, as well as documentation of the child’s medical needs and current treatment, often needed to apply for health and/or welfare services and support.

## Discussion

This study explored family care and communication needs in home-based PPC and highlights the substantial challenges faced by parents of children with LL/LT conditions. Parents felt a lack of comprehensive understanding of their child’s care, impacting the entire family. They emphasized the need for services that alleviate the burden of coordinating hospital consultations and health services. Also, parents warranted a shared EHR to improve communication and information sharing, along with easier ways to contact knowledgeable HCPs regarding their child’s needs.

The parents in our study often felt misunderstood or overlooked, leading to a constant full-time struggle to secure the necessary care and services for their children. This aligns with previous studies indicating that parents reported a negative impact on their quality of life when they felt unacknowledged or disbelieved in interactions with healthcare or social service providers [14]. In municipal settings, parents described that they had to advocate for services due to the limited knowledge of municipal staff related to the comprehensive situation surrounding the ill child’s needs [14]. Lazzarin (2018) found that parents of children with LL/LT conditions spend up to nine hours a day on tasks related to their child’s care, illustrating the complex and time-consuming practical tasks these parents are facing [27]. In the majority of the families we interviewed, there was a sick child with one or more siblings, and the parents openly shared how these siblings were sidelined. These parents struggled with guilt toward their child’s siblings due to a lack of capacity to meet their needs, which is familiar with other studies [14, 28, 29]. A study targeting adult siblings found that having a sibling with an LL/LT condition may have a significant and lasting impact on the adult siblings, and the siblings expressed an unmet need for support, particularly in the form of psychotherapy and peer support [30]. Kirk and Pryjmachuk (2024) highlight the importance of including siblings in PPC and developing family-based interventions and follow-up that also promote sibling–parent relationships [30]. Studies suggest that strengthening the opportunities for parents to meet the needs of siblings can contribute to a more balanced distribution of care and ensure that all family members receive the support they need [14, 20]. Family-centered theory emphasizes the importance of considering the family as a whole in the care process advocating collaborative caregiving, where families work in partnership with HCPs to determine the content of their child’s services [19]. To align with family-centered theory [19], HCPs should strive to understand and address the emotional and practical burdens placed on parents and provide support that enables them to balance their caregiving responsibilities with their own well-being.

Our study found that parents need more support from municipal care coordinators involved in the care of their children. Parents shared stories indicating that coordinators often failed to provide the assistance they considered necessary. In particular, the coordination of consultations and follow-up within specialist healthcare services emerged as a significant bottleneck. A systematic review has similarly found that a lack of support from skilled HCPs with appropriate experience poses a key challenge in the coordination of care for these families [7]. HCPs serving as coordinators face challenges similar to those reported by the parents in our study. These include a lack of experience, insufficient training, and limited time to carry out their responsibilities effectively. Furthermore, the absence of clear job descriptions, standardized procedures, and established routines further complicates their ability to fulfill the coordinator role [31]. Furthermore, coordinators may feel squeezed between obligations toward the families and their needs, and the resources offered by the municipality [31]. The coordinators may not have the mandate to make autonomous decisions in the way that the families require [31]. Consequently, the work of coordinators is conducted in various ways, leading to inconsistency and inefficiency in the services provided. In line with our findings, one approach to improve the situation for families and coordinators may be to strengthen the coordinators’ competencies in coordination and clarify the coordinators’ mandate [31]. Furthermore, the parents in our study expressed the need to establish a more integrated collaboration between municipal services and specialist healthcare service, a recommendation also supported by previous studies [15, 32]. This could contribute to a more cohesive and seamless experience for families, where they do not feel responsible for coordinating complex and time-consuming tasks on their own.

Our study highlighted a need among parents for easier and more direct contact with HCPs in specialist healthcare services. Parents viewed HCPs in specialist healthcare services as an important source of support and information, but they found the existing communication processes complicated and random, which led to frustration and uncertainty. Communication is the very core of family-centered theory [19], and previous research finds that families view availability and communication as the most important issues for collaborative relations with HCPs and healthcare services [7]. In our study, parents expressed a need for dedicated contact persons within specialist healthcare services, such as contact doctors, familiar HCPs, or coordinators who can offer continuous and consistent support. Conversely, parents often felt that HCPs in municipal services lacked the necessary experience and expertise to adequately support their children’s needs, a concern that has also been confirmed in previous studies [15]. This may lead parents to turn to specialist healthcare services for advice and guidance rather than to utilize their primary healthcare services [33, 34]. Furthermore, we found that parents highly value specialist healthcare services, where they are more likely to receive answers to their questions and discuss concerns about their sick child with experienced HCPs. This finding aligns with previous research on communication needs among parents of children with chronic serious illnesses [34, 35]. Having confidence in knowing whom to contact when needed, along with access to HCPs who demonstrate a compassionate and open communication style, such as taking time to listen, answering questions, acknowledging parental views, and showing empathy, is important for parents [9, 35, 36]. However, the parents in our study experienced a great variation in how their contact with HCPs functioned, and parents felt confused about their rights to have a designated contact person within the specialized healthcare services. They also talked about how they dedicated considerable time to gathering and sharing information about their ill child with relevant HCPs. Parents envisioned that a shared EHR, where relevant HCPs had access to read and report, could be one solution to overcome care fragmentation and promote seamless information exchange both in acute and nonacute contacts with HCPs. Conversely, previous research has shown that both parents and HCPs emphasize the importance of information exchange in providing high-quality care for patients with complex needs [37, 38]. Effective communication and information sharing among parents, HCPs, educational staff, and personal assistants could be impacted by the lack of possibilities for interaction and healthcare platforms that do not facilitate efficient information flow [38]. Also, HCPs may be concerned about not having current updates on a child’s condition before home visits [39], e.g., due to the involvement of multiple professionals and the unpredictable nature of the child’s illness trajectory [37, 39]. A shared EHR could allow parents, HCPs, and other professionals involved access to the child’s health data, daily reports, and advance care plans, improving preparedness for home visits as well as unifying various professions and services [39].

The findings underscore the importance of strengthening care coordination, improving communication solutions, and ensuring a comprehensive approach that include support for siblings. Enhancing the competencies and mandates of care coordinators, along with implementing shared EHRs, could foster more comprehensive care for these families. By fostering a collaborative partnership with families, HCPs and the healthcare systems, it could help ensure that the principles of PPC are upheld, ultimately improving the quality of care for children with LL/LT conditions and their families.

### Strengths and limitations

Our study included parents of children with a wide range of LL/LT conditions from all health regions in Norway, representing a diversity of experiences and needs. A total of three fathers participated in the focus groups, which is a strength because fathers have often been underrepresented in PPC studies [40, 41]. We believe the fathers’ participation contributed to a more nuanced and broader picture of the family’s needs and perspectives because they provided unique insights into the emotional and practical challenges faced by families. However, there were too few fathers included to explore potential differences between the fathers and mothers’ descriptions. Based on participants with diverse backgrounds and experiences from various geographical locations who had different levels of access to HCPs and healthcare services, and the participants’ willingness to share a wealth of experiences, the sample size combined with our narrow aim was deemed to have generated sufficient information power [42].

Some of the focus groups consisted of couples participating alongside their partners. This dynamic could be both a strength and a weakness, depending on how the interactions between the participants and within the specific couples unfold. This dynamic may have led to an uneven power distribution within the group; however, we experienced that it created a more comfortable atmosphere where participants felt safe and shared their experiences. Dependability was enhanced by using the same moderator and the same main topics in the interview guide for all interviews. Transferability was enhanced by carefully outlining the study’s context, describing the sample, data collection, and analysis, as well as rich descriptions of findings with quotes. This can enable researchers and HCPs to assess the transferability of our findings to their contexts. However, a limitation of our study is the lack of details on the health and medical conditions of the children in which the parents shared their reflections about. PPC shares many characteristics with complex care, particularly in its emphasis on addressing comprehensive and long-term needs. A family-centered approach is considered essential in PPC, as it is in all contexts where the child is at the center of care, ensuring that interventions are holistic, coordinated, and responsive to the needs of both the child and their family [19].

Our clinical backgrounds facilitated relevant and insightful probing questions during the interviews, enhancing the depth and clarity of the participants’ descriptions. However, our clinical background may have also introduced preconceived notions or led us to overlook certain aspects during the interviews and analysis. To address the issue of preconceptions, we engaged in a reflexive process, actively considering and analyzing our actions, assumptions, and positions throughout the research process, which enhanced the transparency and dependability of the study.

## Conclusion

Parents of children with LL/LT conditions encounter complex and time-consuming caregiving responsibilities, highlighting the need for a coordinator who can manage interactions across the various levels of healthcare services involved in their child’s care. Furthermore, parents suggested that a shared EHR system could improve information sharing and provide easier access to all relevant details about their child. By implementing more comprehensive care, some of the burdens on parents can be alleviated, ensuring that the entire family receives the support they require. Furthermore, these findings have implications for the development of improved information-sharing systems to enhance the experience of home-based PPC.

Future research should focus on care models and healthcare systems that promote easier communication with HCPs and better coordination of healthcare services within PPC. Moreover, future studies should include the perspectives of both the sick child and their siblings to gain a more comprehensive understanding of their needs in the context of home-based care.

## Supplementary Information

Supplementary Material 1.

## Data Availability

The data transcripts generated and analyzed during our study are not publicly available due to privacy regulations. However, interested individuals may request access to the data from the corresponding author upon reasonable request. It is important to note that obtaining the data will require a reasonable request and permission from the Norwegian Agency for Shared Services in Education and Research.
